# The Impact of Long‐Term Tai Chi Practice on the Trust Behavior of Middle‐Aged People

**DOI:** 10.1002/brb3.70254

**Published:** 2025-01-19

**Authors:** Hao Hong, Xin Yu, Qiaoling Li, Bing Li, Panpan Zhang

**Affiliations:** ^1^ Wushu College Henan University Kaifeng China; ^2^ Department of Psychology, Faculty of Education Henan University Kaifeng China

**Keywords:** ERP, middle‐aged people, Tai Chi, trust behavior

## Abstract

**Purpose:**

Trust behavior is of fundamental importance for social stability and development. Middle‐aged people, owing to their abundant social resources and extensive experience, have a significant impact through their trust behavior. However, research on enhancing their trust behavior is relatively scarce. Tai Chi, as an activity that combines physical and mental exercise characteristics, may potentially influence the trust behavior of middle‐aged people, yet direct research in this regard is lacking. The objective of this study is to explore the impact of Tai Chi on the trust behavior of middle‐aged people and its underlying neural mechanisms.

**Method:**

This study recruited two groups of middle‐aged individuals as research participants. Specifically, the Tai Chi group consisted of 37 middle‐aged people with long‐term Tai Chi practice experience, while the control group comprised 29 middle‐aged individuals without any Tai Chi practice background. The behavioral performance and EEG data of the participants in both groups were recorded during their participation in the trust game.

**Results:**

At the behavioral level, the trust rate of the Tai Chi group was higher than the random level (50%), and they made decisions more quickly. The event‐related potential (ERP) data revealed that there were interaction effects between the Tai Chi group and the control group in the P2 and N2 components, which are ERP indicators during the decision‐making stage. Moreover, in the feedback stage, the feedback‐related negativity (FRN) amplitude evoked in the Tai Chi group was smaller than that in the control group.

**Conclusion:**

Long‐term practice of Tai Chi can enhance decision‐making speed and influence brain activity. It provides important reference for understanding the relationship between Tai Chi and the trust behavior of middle‐aged people. Although there are limitations in this study, it lays a foundation for future research.

## Graphical abstract

Long‐term Tai Chi practice can enhance the decision‐making speed of middle‐aged people, and their trust rate is higher than the random level. The electroencephalogram data reveal the relevant neural mechanism. This study provides an important reference for understanding the impact of Tai Chi on the trust behavior of middle‐aged people.

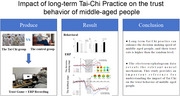

**Abbreviations**
ANOVA: analysis of variance
EEG: electroencephalogram
EOG: electro
ERP: event‐related potential
fMRI: functional magnetic resonance imaging
fMRI: functional magnetic resonance imaging
FRN: feedback‐related negativity
ICA: independent component analysis

## 1 Introduction

Trust is defined as an individual's willingness to hand over personal resources to others and to bear the corresponding risk (Mayer, Davis, and Schoorman 1995; Moretto, Sellitto, and di Pellegrino 2013). It plays a significant role in social interaction and cooperation (Krueger et al. 2008). Trust behavior is akin to prosocial behavior, which can procure economic and social benefits at the lowest cost for both parties (van Houwelingen and van Dijke 2023). In the digital age, the popularization of mobile technology has rendered the environment in which trust behaviors occur even more complex. Mobile applications have become an indispensable part of people's lives, providing new platforms for information dissemination and interaction. However, this also brings challenges such as information overload and misinformation, which may make it more difficult for trust behaviors to emerge. For example, studies have shown that the vast amount of information on mobile applications can lead to confusion and uncertainty, thus affecting users' trust judgments (Muliadi et al. 2024; Pang and Liu 2023; Pang et al., 2024; Pang and Yang 2024; Pang and Zhang 2024). Consequently, enhancing individual trust behavior has emerged as a research topic deserving of attention. Currently, the commonly employed interventions for improving trust behavior encompass cognitive‐behavioral intervention (Bauer, Keusch, and Kreuter 2019), team‐building activities (Ndulue et al. 2012), meditation (Hutcherson, Seppala, and Gross 2008), mindfulness (Berry et al. 2018; Donald et al. 2019), and various forms of exercise (Goudas and Magotsiou 2009). Among these, mindfulness and exercise have garnered more interest due to their favorable intervention effects.

The related research on mindfulness and trust cooperation has substantiated that mindfulness exerts a significant influence on individual altruistic tendencies, prosocial behavior, and trust behavior from the perspectives of intervention duration and specific subject characteristics (Berry et al. 2018; Donald et al. 2019; Jimison 2021; Williams 2013). For instance, certain researchers have discovered that both single‐session mindfulness interventions and long‐term mindfulness programs have an impact on trust behavior (Berry et al. 2018; Donald et al. 2019). Moreover, the enhancement of cooperative behavior through mindfulness was more pronounced among individuals with an initially low willingness to donate (Iwamoto et al. 2020). Recently, some doctoral candidates have utilized the trust game as a research methodology to investigate the impact of mindfulness on trust behavior as a graduation research theme. Their studies have revealed that mindfulness interventions can augment trust behavior. For example, a study employing nuclear magnetic resonance found that mindfulness affects trust behavior by altering the values and cognition of the participants, accompanied by a relative increase in activation within the ventromedial prefrontal cortex (PFC) and orbitofrontal cortex (Williams 2013). Another study demonstrated that mindfulness could even ameliorate an individual's deep‐rooted racial discrimination, thereby increasing trust behavior (Jimison 2021).

The related research on the relationship between exercises and trust cooperation has revealed that both cooperative sports, competitive sports, and other multi‐ or single‐person exercises are conducive to enhancing trust behavior (Goudas and Magotsiou 2009; Hu and Yang 2021; Li et al. 2020). For instance, children with experience in collective movement practice demonstrated better social skills and cooperation (Goudas and Magotsiou 2009). Recently, a study involving a large number of participants (526 individuals) found that long‐term aerobic exercise could improve trust behavior (Hu and Yang 2021). Even competitive sports athletes, who engage in a significant amount of antagonistic behaviors, such as those in basketball and football, also exhibited more cooperative behaviors (Li et al. 2020).

As demonstrated above, both mindfulness and exercise have the potential to enhance trust behavior. It has been observed that the subjects predominantly selected in the aforementioned studies are mostly teenagers. In reality, the cooperation and trust behavior of middle‐aged individuals have a more substantial impact on social development, given their possession of greater social wealth and resources. Consequently, it is more practical to investigate ways to improve the trust behavior of middle‐aged people. For middle‐aged individuals, long‐term systematic mindfulness training or exercises with specific requirements regarding venue, time, and the number of participants, as well as simple repetitive exercises, are often difficult to maintain over an extended period. In contrast, Tai Chi does not necessitate any special clothing, equipment, or specific settings. It exhibits excellent adaptability and can be practiced at any time and place. Tai Chi is not only a form of boxing within Chinese martial arts but also represents an intangible cultural heritage of humanity. Its movements and practice embody profound cultural and philosophical concepts. During the practice of Tai Chi, one not only focuses on the smoothness of body postures and movements but also emphasizes the calmness and concentration of the mind. By regulating breathing and the state of mind to guide the movements, a harmonious unity of the body and mind can be achieved. According to the characteristics of the Tai Chi practice process, it is defined as a mindfulness‐based aerobic exercise in the field of psychology (Posadzki and Jacques 2009), and this definition is supported by the research of Chen et al. Their findings indicated that 8 weeks of Tai Chi practice could have a positive effect on mindfulness levels, and this effect could persist for up to 24 weeks after the conclusion of training (Chen et al. 2021). Evidently, Tai Chi is more engaging than other simple repetitive aerobic exercises. Therefore, it is easier to adhere to in the long term. Moreover, it has a certain impact on the psychological and physical health of practitioners. In recent years, research on the impact of Tai Chi on mental health has become a prominent topic in the field of psychology. The main findings center around the influence of Tai Chi on cognitive function and emotional changes. For example, some researchers have discovered that long‐term practice of Tai Chi can improve cognitive abilities (Walsh et al. 2015), while others have found that Tai Chi is beneficial for alleviating anxiety and depression (X. Liu et al. 2015; Qi et al. 2022).

Currently, the area regarding the influence of Tai Chi on trust behavior remains relatively unexplored. There is a lack of direct studies examining the impact of practicing Tai Chi as an independent variable on trust behavior (the dependent variable). However, some research has delved into the effect of Tai Chi practice on cognitive function and its potential relationship with trust behavior. For instance, Tai Chi exercise has been shown to activate several brain regions, such as the PFC, motor cortex (MC), and occipital cortex (OC) (Robertson and Marino 2015; X. Wang et al. 2023). These regions are crucial for emotion regulation, social cognition, and decision‐making (Z. Liu et al. 2018). Emotion regulation, social cognition, and decision‐making are closely intertwined with the development and maintenance of trust (V. K. Lee and Harris 2013; Song, Colasante, and Malti 2018). Some studies have discovered that the positive effect of Tai Chi on improving emotional health may enhance an individual's capacity for empathy and trust in social situations (X. Liu et al. 2015; Qi et al. 2022). Therefore, to bridge the gap in current research, it is essential to conduct a clear investigation into the direct relationship between Tai Chi and trust behavior.

The classic paradigm for investigating trust behavior is the trust game (Berg, Dickhaut, and Mccabe 1995). In particular, this game is typically carried out by two anonymous players, namely, the trustor and the trustee. Both parties possess a certain amount of money. The trustor has to decide whether to invest a specific sum of money in the other party. Once the investment is made, the amount of money will triple in the hands of the trustee. Subsequently, the trustee is required to determine whether to return a portion of the money to the trustor.

The methods for studying trust behavior encompass questionnaires, behavioral studies, functional magnetic resonance imaging (fMRI), and event‐related potentials (ERP). Among them, ERP technology is widely utilized in the research of trust behavior due to its high temporal resolution. The neural response process of trust behavior is divided into the stages of decision‐making and feedback (Chao et al. 2018; Y. Wang et al. 2017; Yiwen et al. 2015; Y. Wang et al. 2016). They have observed the P2 and N2 components in the decision‐making stage and the feedback‐related negativity (FRN) and P3 components in the feedback stage. Specifically, the decision‐related P2 is a positive component distributed in the middle of the frontal area, reaching its peak at around 150–300 ms. The P2 is associated with risk perception and evaluation in decision‐making scenarios, reflecting the assessment process of decision initiation (Gajewski, Stoerig, and Falkenstein 2008). For instance, in the trust game, a trust choice with higher risk and uncertainty induces a larger P2 amplitude (Yiwen et al. 2015). The decision‐related N2 is a negative component distributed in the middle of the frontal area, peaking within 200–350 ms after the stimulus is presented. The N2 reflects cognitive control and conflict monitoring (Folstein and Van Petten 2008; Schmajuk et al. 2006). For example, in the trust game, distrust elicits a larger N2 amplitude as it violates the reciprocity principle, leading to greater conflict (Chao et al. 2018; Yiwen et al. 2015). In the feedback stage, the FRN peaks approximately within 250–300 ms after the feedback onset and is located in the anterior cingulate cortex (ACC). The FRN reflects a reward prediction error (Sambrook and Goslin 2015). In the trust game, a loss elicits a more negative FRN than a gain (Chao et al. 2018; Y. Wang et al. 2017; Yiwen et al. 2015; Y. Wang et al. 2016). In addition, the P300 is the positive peak at 300–600 ms after feedback onset, which is related to the motivational‐emotional significance of the outcome. A gain with positive and beneficial feedback can induce a larger P3 amplitude than a loss (Guo et al. 2022).

To sum up, trust behavior is of vital importance for social stability and development. The social exchange theory suggests that trust is a psychological inclination that individuals develop based on their expectations of costs and benefits in social exchange relationships (Ahmad et al. 2023). Middle‐aged people, with their rich social resources and experiences, frequently encounter trust decisions in various social exchange contexts such as business cooperation and community mutual assistance. The outcomes of these decisions not only affect their own interests but also have a profound impact on the distribution of social resources and the construction of cooperation networks. The cognitive development theory reveals that although middle‐aged people have mature cognitive abilities, they are prone to forming fixed mindsets, which increases the difficulty in establishing trust. However, research on enhancing their trust behavior is scarce. Existing intervention methods such as cognitive‐behavioral intervention, team‐building activities, and meditation have been attempted, but they have limitations for middle‐aged people. For example, cognitive‐behavioral intervention is time consuming and labor intensive, team‐building activities are restricted by venue and time and have limited participation, and meditation is difficult to maintain in the long term. Tai Chi has unique advantages as its practice is not restricted by venue and time, fitting well into the busy lifestyles of middle‐aged people. Nevertheless, the specific impact and neural mechanism of Tai Chi on the trust behavior of middle‐aged people remain unclear. Therefore, in an attempt to fill the void where there is a lack of direct research on the influence of Tai Chi on the trust behavior of middle‐aged people, we selected two groups of participants: the Tai Chi group and the control group. We employed the trust game and recorded the participants' EEG data during the game. On the basis of previous studies (Chao et al. 2018; Y. Wang et al. 2017; Yiwen et al. 2015; Y. Wang et al. 2016), we chose to observe the P2 and N2 components in the decision‐making stage and the FRN and P3 components in the feedback stage. We hypothesized that differences between the Tai Chi group and the control group could be detected in the behavioral index as well as the related ERP components.

## 2 Method

### 2.1 Participants

Two considerations were taken into account to determine the sample size. Firstly, based on previous research that utilized similar variable manipulation (Xie et al. 2023), we determined the minimum sample size by using G*Power 3.1.9.7 (Faul et al. 2007) and the following thresholds: a medium effect size of *f* = 0.25 (Cohen 2016), *α* = 0.01, 1 − *β* = 0.80, for the interaction of 2 × 2 mixed‐design (with 1 between‐subject factor and 1 within‐subject factor). The results indicated that a minimum of 34 participants were required, that is, 17 individuals in each group. Secondly, prior ERP studies regarding trust behavior usually selected approximately 20 people (Chao et al. 2018; Y. Wang et al. 2017; Yiwen et al. 2015; Y. Wang et al. 2016). On the basis of these two principles, 70 middle‐aged participants were recruited from Kaifeng, Henan. Notably, 4 participants were excluded as they questioned the experimental operation or their ERP data had excessive artifacts and noise. Eventually, the data of 66 participants were included in the final data analysis. For the Tai Chi group, 37 participants with over 7 years of Tai Chi experience were recruited, consisting of 20 men and 17 women with an age range of 49–60 years. For the control group, 29 participants without a long history of exercise were recruited, including 14 men and 15 women with an age range of 47–58 years. Meanwhile, both groups of participants graduated from middle school and none of them were engaged in economic‐ or psychological‐related work. All the participants were right‐handed. There was no history of mental illness, and their vision was normal or corrected to normal. The participants were provided with informed consent prior to the experiment. At the end of the experiment, the participants received payment according to their performance.

### 2.2 Trust Game Task

Recently, the adapted trust game, which is a binary trust game, has been applied to the study of trust behavior (Chao et al. 2018; Y. Wang et al. 2017; Yiwen et al. 2015; Y. Wang et al. 2016). In this game, the trustors cooperate with different individuals in each round. This game can effectively avoid the influence of the feedback from the previous round on the trust decision‐making in the next round and highlight the research focus on trust behavior. According to our research content, we also adopt a binary trust game as the research paradigm in the present study. Specifically, the game comprises two roles, namely, the trustor and the trustee. In the present study, all participants play the role of the trustor. Before the commencement of each round of the game, both the trustor and the trustee receive an initial fund of 10 yuan. First, the trustor needs to decide whether to give all the 10 yuan to the trustee. The trustor has two options. One that the trustor chooses not to give the 10 yuan to the trustee. In this case, the current round of the game is terminated, and both players retain their original 10 yuan. The other option is that the trustor gives the 10 yuan to the trustee. In this situation, the 10 yuan given by the trustor will be tripled to 30 yuan and handed over to the trustee. Subsequently, it is the turn of the trustee to make a choice. The trustee also has two options. One option is to keep all the money to himself/herself. In this case, the trustee gets 40 yuan at the end of the current round, which consists of the 30 yuan given by the trustor and the 10 yuan of the initial amount, while the trustor has no money. The other option is to split the income with the trustor. In this case, at the end of the current round, the trustee divides the total amount of 40 yuan (30 yuan given by the trustor + 10 yuan of the original amount) into two parts and returns one of them (20 yuan) to the trustor. In other words, at the end of this round of the game, both sides of the game receive 20 yuan.

### 2.3 Procedure

This experiment was conducted in the EEG laboratory. Upon entering the laboratory, the participants seated themselves comfortably in a chair approximately 1 m away from the computer screen to take part in the experiment and their EEG activities were recorded with EEG equipment throughout the experiment. Prior to the formal experiment, the participants were presented with the detailed rules of the trust game on the screen. The detailed rules that the participants saw were similar to the trust game task section. It was informed to the participants that during the trust decision‐making process, a specific interface will be displayed on the computer screen, which includes the numbers “1” and “3.” Beneath the numbers “1” and “3” are the words “Keep” and “Invest,” respectively. The participants need to clearly understand that choosing “Invest” means they decide to trust the other party, while choosing “Keep” indicates that they do not trust the other party (the meanings represented by the numbers “1” and “3” will be counterbalanced among the subjects). Participants are required to complete the decision‐making operation by pressing the keys corresponding to the numbers “1” or “3” before the interface disappears. To ensure that the participants had grasped the rules after the introduction, we posed the following three questions: (1) If you opt for distrust, how much money will you obtain? (The answer is 10 yuan). (2) If you choose to trust but the other party decides not to return, how much money will you get? (The answer is 0 yuan). (3) If you choose to trust and the other party opts to return, how much money will you receive? (The answer is 20 yuan). Only when the participants answered these three questions correctly could they take part in the experiment. To make the participants believe that the cooperator was a real person, the participants were informed that we had precollected around 500 choices of trustees in the trust game. Specifically, these trustees were required to complete a single trust game on the network with the experimental assistant. Then the choices were stored in the computer. During each round of the trust game conducted by the EEG participants, the computer randomly selected a trustee's choice from them to complete the current round of the game (Chao et al. 2018; Y. Wang et al. 2017; Yiwen et al. 2015; Y. Wang et al. 2016). In fact, all the choices of the trustees were controlled by the computer program written before the experiment to ensure that the outcome reinforcement rate (the proportion of positive feedback after trust choices) was 50% throughout the entire task.

**FIGURE 1:**
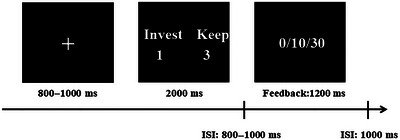
Time sequence of an experimental trial.

Before the formal experiment began, 10 practice trials were set up to help participants become familiar with the whole experimental procedure. The formal experiment consisted of two parts, with a total of 150 trials. Participants could take appropriate breaks and make adjustments between the two parts according to their own conditions to ensure that they could complete the experimental tasks better. As shown in Figure 1, at the start of each trial, a randomly timed fixation point would first appear, with a duration ranging from 800 to 1000 ms. Immediately after that, the decision‐making choice page would be displayed for 2000 ms. During this period, participants had to make a choice of trust or distrust within 2000 ms. If the decision time exceeded 2000 ms, the corresponding data would be regarded as invalid. On this page, participants could choose the key “1” which represented trust or the key “3” which represented distrust, and the meanings of the keys were counterbalanced among the participants. After making the choice, a randomly timed blank screen would appear, also with a duration ranging from 800 to 1000 ms. Then, a feedback page lasting 1200 ms would follow, and there were three possible feedback results: 0, 10, and 30. At the end of each trial, a blank screen lasting 1000 ms would also be presented. The above was the complete step‐by‐step process of one trial (see Figure 1).

### 2.4 EEG Recording and Data Analysis

The participants' brain electrical activities were captured by using a 32‐channel electrode cap, with the distribution of electrode sites in accordance with the modified 10–20 system EEG (Neuroscan Inc., Victoria, Australia). The ground electrode was positioned in the middle above the forehead between FP1 and FP2. The FCz served as the location of the online reference electrode. To monitor eye movements and blinks, the electrooculogram (EOG) was recorded from the electrode beneath the right eye. There was a sampling rate of 1000 Hz and the electrode impedances were maintained at less than 10 kΩ.

After the completion of EEG data collection, we employed the toolbox EEGLAB 2019 (https://vs.vs.sccn.ucsd.eduvs.eeglab) (Delorme and Makeig 2004) and MATLAB's built‐in scripting capabilities for offline analysis. Firstly, the continuous data were offline referenced using the average of TP9 and TP10. Secondly, a zero‐phase, noncausal digital filter with a half‐amplitude cutoff at specific frequencies (−6 dB) was applied, along with a 0.1–30‐Hz high‐pass filter and low‐pass filter using EEGLAB's “basic FIR filter (new, default)” option. Subsequently, the continuous EEG data were segmented from two distinct time windows. The analysis time course ranged from 200 ms before the appearance of the decision‐making interface and the feedback interface to 1000 ms after their appearance. Subsequently, a baseline correction was carried out 200 ms prior to the onset of the related pages, respectively. We eliminated artifacts related to muscular and ocular activities by utilizing the IClabel tool and the Infomax independent component analysis (ICA) algorithm (runica). According to the previous study (Pion‐Tonachini, Kreutz‐Delgado, and Makeig 2019), the IClabel tool adopts a machine‐learning‐based methodology to automatically label the independent components based on their spatial and temporal characteristics. Any segments with amplitudes exceeding ±80 µV were removed as additional artifacts or noise. After a series of operations, there were still approximately 30 trials for each participant under the conditions to be analyzed. Finally, the ERP components corresponding to the participants' trust choices and distrust choices were, respectively, superimposed and averaged as well as grand averaged. Meanwhile, the ERP components induced by the gain feedback and loss feedback after the participants' trust choices were also, respectively, superimposed and averaged as well as grand averaged.

In this study, we adhered to two criteria for selecting the observed ERP components as well as their corresponding time windows and electrode sites. Firstly, the existing related research determined which ERP components to observe and how to select their time windows and electrode sites (Chao et al. 2018; Y. Wang et al. 2017; Yiwen et al. 2015; Y. Wang et al. 2016). Secondly, we were able to choose the appropriate time window and the electrode site with the maximum activation by observing the average waveform and topographic map obtained in this study. Hence, we observed the P2 and N2 components in the decision‐making stage. Specifically, for the P2 component, the average amplitude was calculated at the Fz electrode site, with a time window of 150–250 ms (Chao et al. 2018; Y. Wang et al. 2017; Yiwen et al. 2015; Y. Wang et al. 2016). As for the N2 component, the average amplitude was also calculated at the Fz electrode site, with a time window of 250–350 ms (Chao et al. 2018; Y. Wang et al. 2017; Yiwen et al. 2015; Y. Wang et al. 2016). In addition, we observed the FRN and P3 components in the feedback stage. Specifically, for the FRN component, the average amplitude was calculated at the Fz electrode site, with a time window of 200–300 ms (Y. Wang et al. 2017; Yiwen et al. 2015; Y. Wang et al. 2016). For the P3 component, the average amplitude was calculated at the Pz electrode site, with a time window of 300–600 ms (Yiwen et al. 2015).

### 2.5 Statistical Analysis

Based on previous studies (Chao et al. 2018; Y. Wang et al. 2017; Yiwen et al. 2015; Y. Wang et al. 2016), trust rate and reaction time were selected as behavioral indicators. Specifically, the trust rate was defined as the ratio of the number of trust choices made by participants to the total number of decision‐making instances. Reaction time was defined as the time needed for a participant to make a choice after the appearance of the decision choices page. Firstly, we conducted an independent‐sample *t*‐test on the trust rate and reaction time of the two groups of participants with different genders to observe whether gender would have an impact on trust behavior in the present study. Secondly, for the behavioral data, an independent‐sample *t*‐test was performed on the trust rate and reaction time. Besides, a single‐sample *t*‐test was utilized to compare the investment rate of the Tai Chi group with a random level of 50%. Similarly, a single‐sample *t*‐test was also employed to compare the investment rate of the control group with a random level of 50%. Finally, for the ERP data, SPSS25.0 was used to conduct a 2 (group: Tai Chi vs. control) × 2 (decision choices: trust vs. distrust) mixed analysis of variance (ANOVA) on the mean amplitudes of the P2 and N2 components. Similarly, 2 (group: Tai Chi vs. control) × 2 (feedback: gain vs. loss) mixed ANOVA was conducted on the mean amplitudes of the FRN and P3 components. To standardize the reporting of statistical results, we used the Greenhouse–Geisser method to correct degrees of freedom when necessary. The Bonferroni correction method was adopted to adjust the post hoc tests of significant main effects. Simple‐effect analysis was used to further explore significant interactions. The partial eta‐squared (*η*2 *p*) was provided in all results, which serves as a measure of the proportion of variance, indicating the percentage of variation accounted for by the independent variable.

## 3 Result

### 3.1 Behavioral Results

**FIGURE 2:**
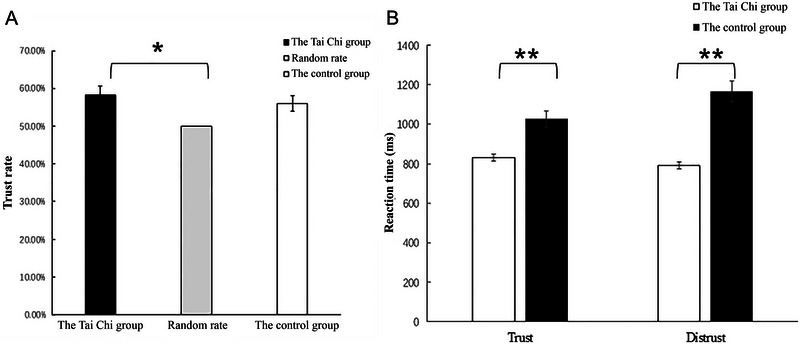
Error bars represent the standard error of the mean. *p < 0.05; ** *p* < 0.01.

The independent‐sample *t*‐test demonstrated that there was no significant gender difference in trust rate and reaction time within both the Tai Chi group and the control group (*p* > 0.05). The average trust rate of the Tai Chi group was 58.22%, and that of the control group was 56%. The independent‐sample *t*‐test conducted on the trust rates of the two groups revealed that there was no difference in the trust rate between the Tai Chi group and the control group (*t*(64) = −0.397, *p* = 0.271) (see Figure 2A). Through the single‐sample *t*‐test, the trust rate of the Tai Chi group was significantly higher than the random level (50%) (*t*(36) = 2.066, *p* = 0.046), while there was no significant difference between the trust rate of the control group and the random level (*p* > 0.05). Regarding the reaction time, the Tai Chi group's reaction time was significantly shorter than that of the control group, regardless of whether the participants chose trust (*t*(64) = 2.515, *p* = 0.005) or distrust (*t*(64) = 4.082, *p* < 0.001) (see Figure 2B).

### 3.2 ERP Results

#### 3.2.1 The Decision‐Making Stage

A 2 (group: Tai Chi vs. control) × 2 (decision choices: trust vs. distrust) mixed ANOVA was conducted on the P2 mean amplitudes of the Fz electrode site. A main effect of decision was significant (*F*(1, 64) = 5.015, *p* = 0.029, *η*2 *p* = 0.081), suggesting that trust (*M* = 1.561, SE = 0.398) elicited a larger P2 amplitude than distrust (*M* = 0.806, SE = 0.306). We also observed a significant main effect of group (*F*(1, 64) = 16.488, *p* < 0.001, *η*2 *p* = 0.224), with a larger P2 amplitude for the Tai Chi group (*M* = 2.452, SE = 0.398) compared to the control group (*M* = −0.085, SE = 0.481). In addition, the interaction between the decision and group was significant (*F*(1, 64) = 3.782, *p* = 0.057, *η*2 *p* = 0.062). The simple‐effect analyses demonstrated that the P2 for the control group was larger in trust (*M* = 0.621, SE = 0.613) than distrust (*M* = −0.791, SE = 0.472) (*F*(1, 64) = 7.378, *p* = 0.009, *η*2 *p* = 0.115), but the P2 for the Tai Chi group has no decision difference (*F*(1, 64) = 0.053, *p* = 0.818, *η*2 *p* = 0.001).

**FIGURE 3:**
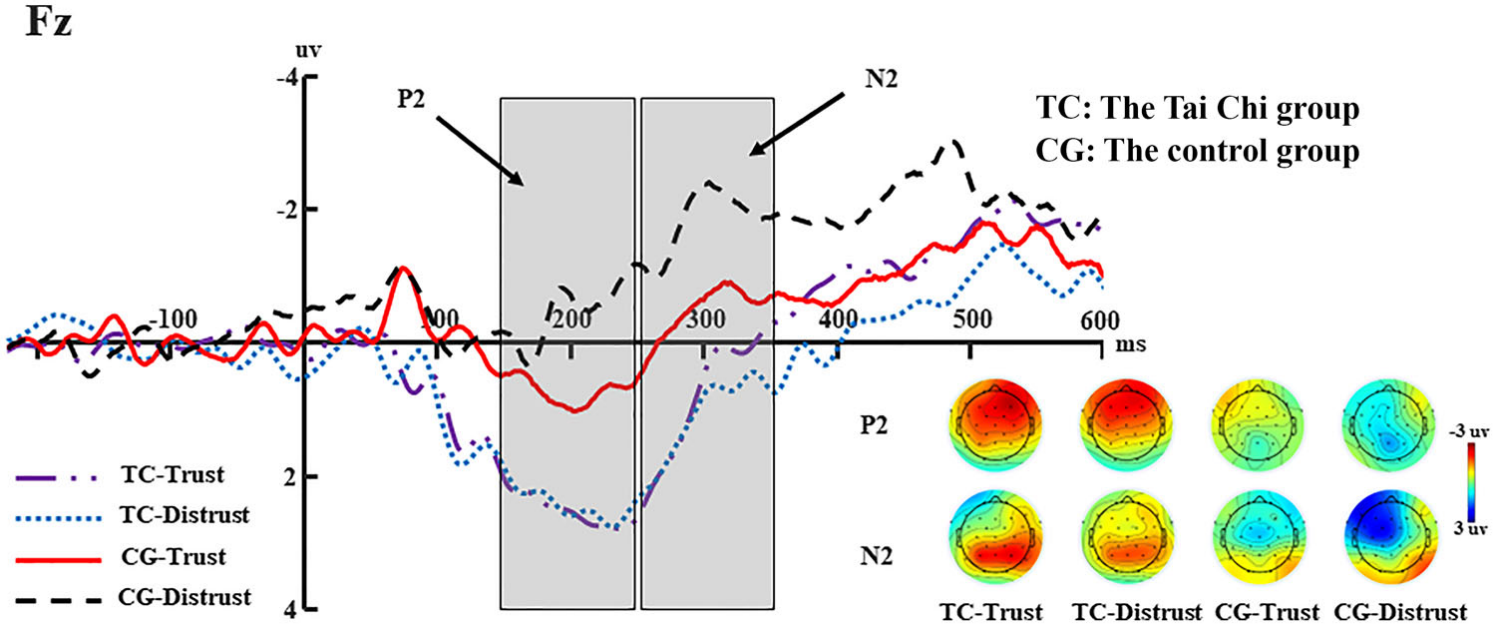
Grand‐averaged waveforms, topographic maps, and amplitudes of the P2 (150–250 ms) and N2 (250–350 ms) averaged at the Fz electrode for trusting and distrusting decision choices, separated by the Tai Chi group and the control group. The gray area highlights the time window.

A 2 (group: Tai Chi vs. control) × 2 (decision choices: trust vs. distrust) mixed ANOVA was conducted on the N2 mean amplitudes of the Fz electrode site. A main effect of group was significant (*F*(1, 64) = 4.998, *p* = 0.029, *η*2 *p* = 0.081), suggesting that the control group (*M* = −1.347, SE = 0.613) elicited more negative N2 amplitude than the Tai Chi group (*M* = 0.433, SE = 0.508). And the interaction between the decision and group was significant (*F*(1, 64) = 4.632, *p* = 0.036, *η*2 *p* = 0.075). The simple‐effect analyses demonstrated that the N2 for the control group was more negative in distrust (*M* = −2.029, SE = 0.706) than trust (*M* = −0.666, SE = 0.691) (*F*(1, 64) = 4.153, *p* = 0.046, *η*2 *p* = 0.068), but the N2 for the Tai Chi group has no decision difference (*F*(1, 64) = 0.834, *p* = 0.365, *η*2 *p* = 0.014). The main effect of decision was not significant (*F*(1, 64) = 0.974, *p* = 0.328, *η*2 *p* = 0.017) (see Figure 3).

#### 3.2.2 The Feedback Stage

**FIGURE 4:**
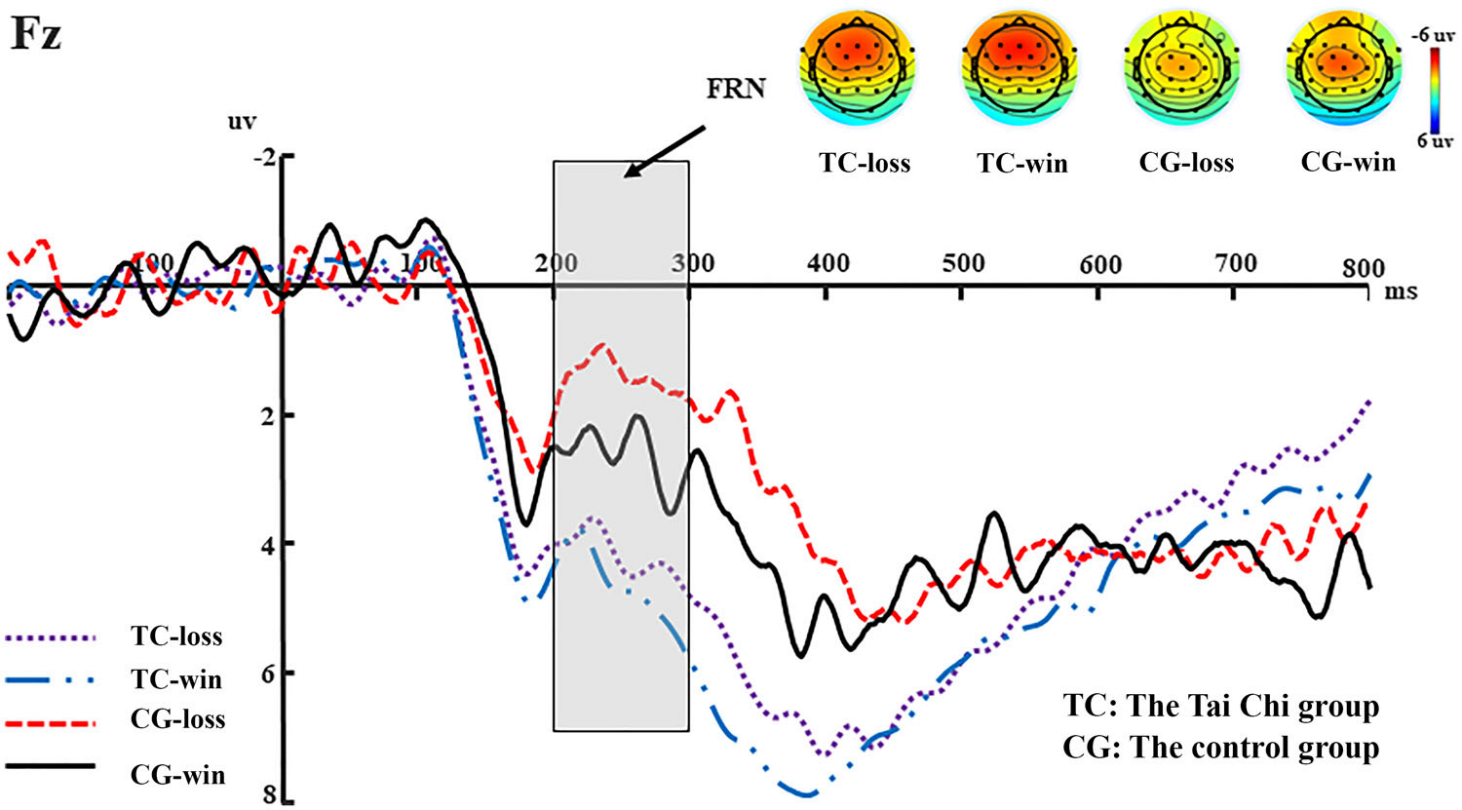
Grand‐averaged waveforms, topographic maps, and amplitudes of the FRN (200–300 ms) averaged at the Fz electrode for losses and gains, separated by the Tai Chi group and the control group. The gray area highlights the time window.

A 2 (group: Tai Chi vs. control) × 2 (feedback: gain vs. loss) mixed ANOVA was conducted on the FRN mean amplitudes of the Fz electrode site. A main effect of feedback was significant (*F*(1, 64) = 4.884, *p* = 0.031, *η*2 *p* = 0.079), suggesting that loss (*M* = 2.850, SE = 0.363) elicited more negative FRN amplitude than gain (*M* = 3.499, SE = 0.444). The main effect of the group was significant (*F*(1, 64) = 8.319, *p* = 0.006, *η*2 *p* = 0.127), suggesting that the control group (*M* = 2.084, SE = 0.583) elicited more negative FRN amplitude than the Tai Chi group (*M* = 4.265, SE = 0.482). However, the interaction between feedback and group was not significant (*F*(1, 64) = 0.931, *p* = 0.339, *η*2 *p* = 0.016) (see Figure 4).

**FIGURE 5:**
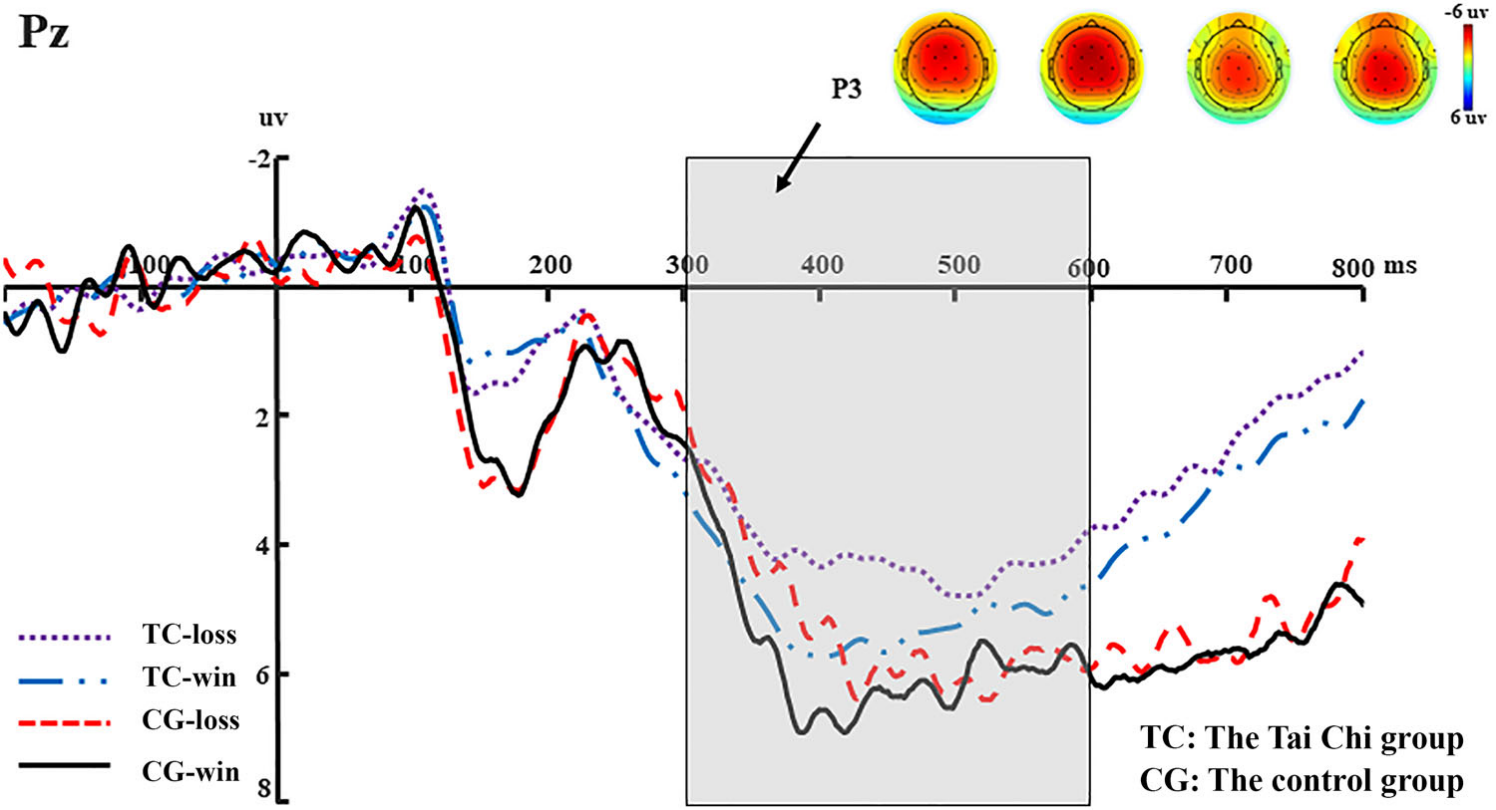
Grand‐averaged waveforms, topographic maps, and amplitudes of the P3 (300–600 ms) averaged at the P3 electrode for losses and gains, separated by the Tai Chi group and the control group. The gray area highlights the time window.

A 2 (group: Tai Chi vs. control) × 2 (feedback: gain vs. loss) mixed ANOVA was conducted on the P3 mean amplitudes of the Pz electrode site. A main effect of feedback was significant (*F*(1, 64) = 5.592, *p* = 0.021, *η*2 *p* = 0.089), suggesting that gain (*M* = 5.378, SE = 0.577) elicited a larger P3 amplitude than loss (*M* = 4.665, SE = 0.515). However, the main effect of group (*F*(1, 64) = 0.751, *p* = 0.390, *η*2 *p* = 0.013) and the interaction between feedback and group were not significant (*F*(1, 64) = 0.539, *p* = 0.466, *η*2 *p* = 0.009) (see Figure 5).

## 4 Discussion

Middle‐aged people possess higher social status and more resources, and their trust behavior may involve more economic transactions as well as deeper social cooperation. How to enhance the trust behavior of middle‐aged people is an important issue that is beneficial to group interests and social development. Tai Chi appears to be more suitable for intervening in trust behavior compared to mindfulness and regular exercise, as it combines the characteristics of both mindfulness and aerobic exercise and is more interesting and flexible in terms of time and place. However, there is currently no direct evidence to prove that Tai Chi has an impact on trust behavior, and its underlying neural mechanism remains unclear. Therefore, our study aims to observe whether Tai Chi influences the trust behavior of middle‐aged people by combining the ERP technique with the trust game. We divided the participants into the Tai Chi group and the control group according to whether they had long‐term Tai Chi practice experience. Finally, we observed the differences between the two groups, both in terms of behavioral data and ERP data.

Behaviorally, the trust rate of the Tai Chi group was significantly higher than the random level (50%), while no significant difference was observed between the control group and the random level. However, there was no difference in the trust rate between the Tai Chi group and the control group. This might be due to the fact that our study adopted a binary trust game, in which participants only had to choose whether to invest the entire amount rather than any amount of investment, resulting in the possible significant statistical relationship being obscured. Moreover, we observed that the time spent on decision‐making in the Tai Chi group was significantly shorter than that in the control group, which indicated that the Tai Chi group made decisions more quickly. This could be because there is at least a 7‐year difference in exercise experience between the Tai Chi group and the control group, which may lead to differences in individual physical health status. A study found that more active or healthier individuals could allocate more attention resources to the current environment and thus help deal with existing tasks or emergencies (Gomez‐Pinilla and Hillman 2013; Ligeza et al. 2023).

In terms of the electrophysiological results during the decision‐making stage, we observed that the P2 amplitude evoked in the Tai Chi group was significantly larger than that in the control group. Further analysis revealed that in the Tai Chi group, there was no significant difference in the P2 amplitude evoked by the trust and distrust options, whereas in the control group, the P2 amplitude evoked by the trust option was significantly larger than that by the distrust option. According to previous studies, during the decision‐making process of the trust game, the P2 component is closely related to risk assessment and the evaluation process of decision initiation. On the basis of this, we make the following reasonable speculations about the results: Tai Chi, as an aerobic exercise, can prompt the allocation of more attentional resources to the current environment, thereby processing relevant information more efficiently and responding to challenges quickly (Gajewski, Stoerig, and Falkenstein 2008; Yiwen et al. 2015). At the same time, this result may imply that the subjects in the Tai Chi group did not perceive the trust and distrust options as options with different levels of risk. In contrast, the subjects in the control group believed that the trust option carried the risk of being betrayed and was of a higher risk compared to the distrust option. It is human instinct to seek advantages and avoid disadvantages (Müller, Kals, and Maes 2008). Therefore, we infer that the Tai Chi group is more likely to exhibit trust behavior compared to the control group. Furthermore, when observing another component N2 during this stage, we found that the N2 amplitude evoked in the control group was more negative than that in the Tai Chi group. Moreover, in the control group, the N2 amplitude evoked by the distrust option was significantly larger than that by the trust option, while in the Tai Chi group, there was no significant difference in the N2 amplitude evoked by the distrust and trust options. According to previous studies, during the decision‐making process of the trust game, N2 is mainly involved in the cognitive control process and is highly sensitive to conflict detection and response inhibition (Folstein and Van Petten 2008; Schmajuk et al. 2006). On the basis of this, we make the following reasonable speculations about the results: The control group exhibited more cognitive conflict, perhaps because the distrust option violated the reciprocity rule and individuals in the control group needed to invest more cognitive effort to inhibit their distrust behavior and thus make behavior that conformed to social norms. In contrast, the Tai Chi group could exhibit trust behavior that was much higher than the random level in the trust game paradigm of this study without excessive cognitive control. This might imply that individuals who have practiced Tai Chi for a long time have a higher altruistic motivation and a tendency for helping behavior and can exhibit more trust behavior with less cognitive control.

In the feedback stage, the amplitudes of FRN elicited by loss were larger than those by gain. This result was in line with previous studies (Chao et al. 2018; Y. Wang et al. 2017; Yiwen et al. 2015; Y. Wang et al. 2016). The FRN reflects a reward prediction error (Sambrook and Goslin 2015). Specifically, the actual outcome of loss was contrary to the expected gain feedback after the participant made a trust choice, resulting in a larger FRN. Interestingly, we also found that the control group elicited a larger FRN amplitude than the Tai Chi group. Previous studies have found that middle‐aged people are more averse to betrayal (Hong and Bohnet 2007), which may mean that they have a higher expectation of obtaining positive feedback from cooperators after choosing trust. In addition, long‐term practice of Tai Chi, which includes mindfulness components, can promote individual altruism and altruistic behavior (Alarcon and Jessup 2023; Guillou, Grandin, and Chevallier 2021; Kong and Yao 2019; N. C. Lee, Jolles, and Krabbendam 2016; Sijtsma et al. 2023), that is, simply increasing the income of others. In other words, people with more altruistic behavior are less likely to expect feedback from others. Thus, a difference in FRN amplitude exists between the Tai Chi group and the control group. Moreover, we observed that gain induced a larger P3 amplitude than loss. This result was consistent with previous studies because a larger P3 is elicited by positive and beneficial feedback (Guo et al. 2022).

## 5 Conclusions and Limitations

This study integrated behavioral and ERP data to explore the impact of long‐term Tai Chi practice on the trust behaviors of middle‐aged people. In terms of behavior, the trust rate of the Tai Chi group was higher than the random level and they made decisions more quickly. Although there was no significant difference in the trust rate compared with the control group, this trend was still valuable. The ERP data revealed its underlying mechanisms. The differences in the P2 and N2 components during the decision‐making stage between the Tai Chi group and the control group indicated that long‐term Tai Chi practice had changed the brain's cognitive processing of the risks associated with trust behaviors, making people less likely to regard trust as a high‐risk behavior easily and probably increasing the tendency to trust others. Moreover, long‐term Tai Chi practice improved the level of mindfulness, balanced the cognitive control requirements for trust and distrust decisions, and helped to stabilize the tendency of trust behaviors. During the feedback stage, the FRN component showed that the Tai Chi group paid less attention to personal gains and losses and had a stronger altruistic tendency. This also indirectly reflected the positive impact of long‐term Tai Chi practice on trust behaviors, enabling people not to be overly concerned about gains and losses in trust interactions and facilitating the maintenance of trust relationships. This study focused on the middle‐aged population. Although the direct differences in trust behaviors did not reach significance, it deeply analyzed the relationship between long‐term Tai Chi practice and trust behaviors from the perspective of brain mechanisms, providing an important reference for follow‐up studies.

However, there are certain limitations in this study. Firstly, due to the difficulty in recruiting special participants, this study was neither unable to directly compare the effects of mindfulness, exercise, and Tai Chi on trust behavior nor could it determine which intervention method was more beneficial for improving the trust behavior of middle‐aged people. Secondly, this study is a cross‐sectional study. Future longitudinal research could be carried out to explore the stage characteristics of the influence of practicing Tai Chi on trust behavior. In addition, the advantage of ERP technology lies in its precise timing, but it cannot observe the differences in brain region activation among different groups. In the future, fMRI technology can be utilized to observe the brain activation status of middle‐aged people who have been practicing Tai Chi for a long time when making trust decisions. Finally, there will be an increase in the flexibility of the amount of money that participants choose to invest in the trust game to better observe the differences in trust rate.

## Author Contributions

**Hao Hong**: Conceptualization, investigation, supervision, funding acquisition, resources, project administration, writing–review and editing. **Xin Yu**: Conceptualization, methodology, writing–review and editing, data curation, software, investigation, project administration, validation, visualization, formal analysis, writing–original draft, resources. **Qiaoling Li**: Conceptualization, methodology, investigation, supervision, resources, project administration, writing–review and editing. **Bing Li**: Methodology, investigation, data curation, writing–review and editing. **Panpan Zhang**: Methodology, investigation, data curation, writing–review and editing.

## Consent

Informed consent was obtained from all the subjects participating in the study.

## Conflicts of Interest

The authors declare no conflicts of interest.

### Peer Review

The peer review history for this article is available at https://publons.com/publon/10.1002/brb3.70254.

## Data Availability

The data that support the findings of this study are available on request from the corresponding author. The data are not publicly available due to privacy or ethical restrictions.
